# MRI-Based Evaluation of the Flexor Digitorum Superficialis Anatomy: Investigating the Prevalence and Morphometry of the “Chiasma Antebrachii”

**DOI:** 10.3390/diagnostics13142406

**Published:** 2023-07-19

**Authors:** Clara Elsner, Andreas Steven Kunz, Nicole Wagner, Henner Huflage, Stefan Hübner, Karsten Sebastian Luetkens, Thorsten Alexander Bley, Rainer Schmitt, Süleyman Ergün, Jan-Peter Grunz

**Affiliations:** 1 Department of Diagnostic and Interventional Radiology, University Hospital Würzburg, Oberdürrbacher Str. 6, 97080 Würzburg, Germany; 2 Institute of Anatomy and Cell Biology, University of Würzburg, Koellikerstr. 6, 97070 Würzburg, Germany; 3 Department of Radiology, University Hospital, LMU Munich, Ziemessenstraße 6, 80336 Munich, Germany

**Keywords:** flexor digitorum superficialis, flexor tendon, chiasma antebrachii, magnetic resonance imaging

## Abstract

Recent dissection studies resulted in the introduction of the term “chiasma antebrachii”, which represents an intersection of the flexor digitorum superficialis (FDS) tendons for digits 2 and 3 in the distal third of the forearm. This retrospective investigation aimed to provide an MRI-based morphologic analysis of the chiasma antebrachii. In 89 patients (41 women, 39.3 ± 21.3 years), MRI examinations of the forearm (2010–2021) were reviewed by two radiologists, who evaluated all studies for the presence and length of the chiasma as well as its distance from the distal radioulnar and elbow joint. The chiasma antebrachii was identified in the distal third of the forearm in 88 patients (98.9%), while one intersection was located more proximally in the middle part. The chiasma had a median length of 28 mm (interquartile range: 24–35 mm). Its distances to the distal radioulnar and elbow joint were 16 mm (8–25 mm) and 215 mm (187–227 mm), respectively. T1-weighted post-contrast sequences were found to be superior to T2- or proton-density-weighted sequences in 71 cases (79.8%). To conclude, the chiasma antebrachii is part of the standard FDS anatomy. Knowledge of its morphology is important, e.g., in targeted injections of therapeutics or reconstructive surgery.

## 1. Introduction

The flexor digitorum superficialis (FDS) is the largest among the superficial flexor muscles situated in the anterior compartment of the forearm and plays a pivotal role in finger flexion, enabling essential hand movements for daily activities [1]. It is known for its complex structure with many anatomical variances (e.g., anomalous muscle bellies, anomalous tendon arrangement, or intermediate tendons) that are still the subject of ongoing research [2,3]. In 1999, the research group of Elliot et al. presented a novel classification of FDS anomalies and divided them into five types [4]. Type I anomalies involve the attachment of one FDS tendon to another, while Type II anomalies demonstrate the attachment of the FDS tendon to the flexor retinaculum. Type III anomalies occur when the FDS tendon contains a digastric muscle, whereas Type IV anomalies entail a distal extension of the FDS muscle belly. Finally, Type V encompasses anomalies of the FDS muscle in the forearm itself [4]. Among these variations, especially the FDS of the fifth digit has shown significant structural variability, with reports of complete absence in up to 21% of as well as right- and left-hand asymmetry in approximately 26% of cases [5,6,7]. The exact origin of the many muscular varieties is a subject of continued investigation; however, some authors postulate that they represent deviations from individual ontogenesis [8,9]. Embryologic studies suggest that the FDS arises in an initially digastric configuration from a proximal muscular blastema in the carpal region and a distal blastema at the elbow level [9]. From the proximal site, the muscular venter increases gradually into the forearm and distally forms separate muscular flaps that attach to the palmar surfaces of the flexor digitorum profundus (FDP) tendons [8]. It is postulated that an aberrant differentiation of this insertion during embryonic development may account for an FDS variation with an absence of individual tendons [8]. Also, the size of the muscular venter of the FDS depends on the individual extent of muscular blastema shifting during the embryonal period [8].

The clinical significance of the many different anatomic variations of the FDS includes not only atypical findings in physical examinations but also altered use patterns in the injured state, the imitation of soft tissue tumors, and the cause of nerve compression syndromes [3,10,11]. Especially the latter phenomenon has been the subject of many case reports in the literature, which describe anomalous muscle bellies of the FDS passing through the carpal tunnel into the palm, resulting in median nerve irritation and compression [11,12]. With regard to traumatic injuries, it is known that the flexor tendons of the hand often require surgical treatment for the best functional outcome: partial tendon lacerations <50% are commonly managed with debridement alone, partial lacerations >50% may be treated with epitendinous repair, and complete lacerations commonly require multi-strand core repair or even tendon graft surgery [13,14]. Therefore, a detailed understanding of FDS variability is crucial when performing procedures involving the palmar side of the forearm, and the complexity of muscle anatomy should raise the watchfulness for unexpected findings at surgery [3,13].

The gross structure of the FDS is described as follows in anatomical textbooks: In the distal forearm, it is situated underneath the palmaris longus, flexor carpi radialis, and flexor carpi ulnaris muscles and superficial to the flexor digitorum profundus and flexor pollicis longus. At its proximal origin, the FDS arises by the three heads-humeral (from the medial epicondyle of the humerus), ulnar (from the medial coronoid process), and radial (from the oblique line of the radius). Its muscle belly can be separated into a superficial and a deep layer, the former dividing into two parts concluding in tendons for the third and fourth digits, the latter forming an intermediate tendon in the middle of the forearm that transitions into the distal belly for the second digit. The FDS belly for the fifth digit takes its origin from the intermediate tendon and runs in a slightly oblique course to the ulnar side of the distal forearm [1,2].

While the concept of the FDS/2 tendon crossing under the FDS/3 tendon was first described by von Luschka in 1865, this knowledge was largely forgotten until recently [15]. In 2023, Ergün et al. provided a detailed topographical description of the bellies and tendons of the FDS based on anatomical dissection studies. Hereby, the authors demonstrated that the tendon for digit 2 runs from the proximal-ulnar to the distal-radial and while doing so, crosses under the third digit’s FDS tendon from the ulnar to the radial side. They coined this intersection the “chiasma antebrachii”. Moreover, the authors provided the first graphical representation of the FDS that enabled a new view of this muscle [16]. Despite the establishment of the term “chiasma antebrachii” as an anatomical reference, it remains unclear whether this tendon intersection represents the standard anatomy or, rather, an uncommon variant. In addition, it is well known that formalin fixation—which was used for the conservation of cadaveric specimens in the published dissection study—has a deteriorating effect on the quality of organic tissues over time, presumably limiting the reliability of measurements. As reported by Turunen et al., collagen shrinkage, loss of alignment, and spatial variation may result from the fixation process [17]. Further substantiating this assumption, Docquier et al. described that formalin fixation led to slight muscle expansion, fatty tissue shrinkage, and overall flattening of specimens [18]. Therefore, an MRI-based in vivo study on actual patients may allow for a more precise analysis of the anatomical structures and their detailed relations in the distal forearm.

The overall objective of the current study was to investigate the presence and detailed morphology of the chiasma antebrachii through MRI analysis of the forearm in a clinical patient sample, thereby contributing to a better understanding of the variations of the FDS and potentially aiding clinical interventions.

## 2. Material and Methods

### 2.1. Study Population

**Figure 1:**
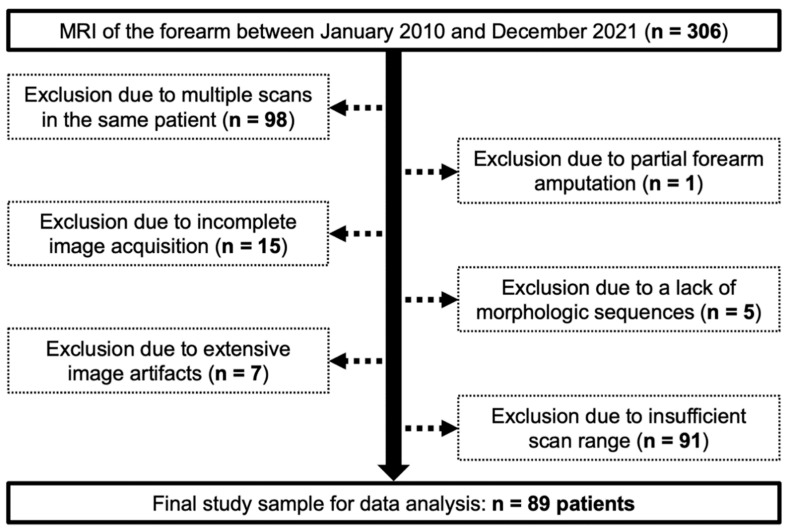
Flow chart for visualization of the study population and exclusion/inclusion criteria.

The local institutional review board approved this retrospective data analysis and waived the need for written informed consent (reference number: 20220818 01). Between January 2010 and December 2021, 306 consecutive MRI examinations of the forearm were available for radiological assessment. Of these, 217 had to be excluded from the data analysis for one of the following reasons: Multiple scans in the same patient (*n* = 98), partial forearm amputation (*n* = 1), incomplete image acquisition (*n* = 15), lack of morphologic T1- and/or T2-weighted sequences (*n* = 5), extensive image artifacts with resulting loss of diagnostic accessibility (*n* = 7) or insufficient scan range to evaluate the entire FDS anatomy (*n* = 91). Summarizing exclusions and inclusions, a flow chart illustrates the population analyzed in this study (Figure 1).

### 2.2. MRI Examinations

Forearm studies were performed both on 1.5 T scanners (Magnetom Aera, Avanto or Symphony, Siemens Healthineers) in 43 scans (48.3%) and 3.0 T scanners (Magnetom Prisma or Skyra, Siemens Healthineers) in 46 scans (51.7%). All examinations were conducted in accordance with the clinically established scan protocol for the respective imaging task, including intravenous contrast application if necessary. Patients adopted a pronated position of the hand in 82 scans (92.1%), a neutral position in 5 scans (5.6%), and a supinated position in 2 scans (2.2%).

### 2.3. Image Assessment

Two radiologists with one and six years of musculoskeletal imaging experience (C.E., J.-P.G.) collectively analyzed the above-described datasets in chronological order using a commercially available picture archiving and communication system (Merlin, Phönix-PACS) installed on a radiological workstation with a certified diagnostic monitor. The observers were given three tasks for their reads: First, to evaluate the presence of the chiasma antebrachii in a dichotomous fashion (0 = no chiasma antebrachii, 1 = identifiable chiasma antebrachii). Second, if applicable, to assess the length of the chiasma. Third, to analyze the position of the FDS/2 and FDS/3 intersection by measuring the distance from the proximal margin of the identified chiasma to the distal radioulnar joint as well as to the elbow joint. Both distinct landmarks were chosen to ensure the reproducibility of measurements. Before commencing their reads, the radiologists reviewed five training cases not included in the study sample.

### 2.4. Dissected Human Forearms

In order to verify the image findings in anatomical dissection studies, the forearms of 11 formalin-fixated body donors from the local anatomical institute (including 6 women, age range: 55–100 years) were assessed. Before their dissection, cadaveric specimens had been fixated by intra-arterial perfusion with 4–4.5% formaldehyde solution, containing formaldehyde 37% (1.2–1.8 L, depending on size and weight of the donors), Carlsbad salt (400 g), chloral hydrate (400 mL), and Lysoformin^®^ (400 mL) in 8 L of water for several hours. Subsequently, the body donors were stored in ethanol-(33%)-solution-filled metal containers for preservation by ethanol vapor for 1–2 years. The use of human material was in full compliance with the university policy for the use of body donors and recognizable body parts.

### 2.5. Statistics

Data analysis was supported by dedicated statistical software (SPSS version 28.0, IBM). The normal distribution of continuous items was analyzed with Kolmogorov–Smirnov tests [19]. For parametric data with normal distribution, mean ± standard deviation is presented; otherwise, we report median and interquartile range values (IQR). Irrespective of the item scale, absolute and relative frequencies are indicated for all variables.

## 3. Results

### 3.1. Study Population

Adhering to inclusion and exclusion criteria depicted in Figure 1, the final study group was comprised of 89 patients, including 41 women (46.1%), and had a mean age of 39.3 ± 21.3 years (range 2–84 years). In 47 cases (52.8%), the left forearm was scanned. MRI examinations were performed for different clinical indications: primary or follow-up imaging of a benign or malignant tumor of the forearm (*n* = 25), a history of trauma (*n* = 35), diagnostic assessment of soft tissue inflammation (*n* = 20), and others, such as pain without trauma history or suspected neuropathy of the median nerve (*n* = 9).

### 3.2. MRI-Based Flexor Digitorum Superficialis Assessment

**Figure 2:**
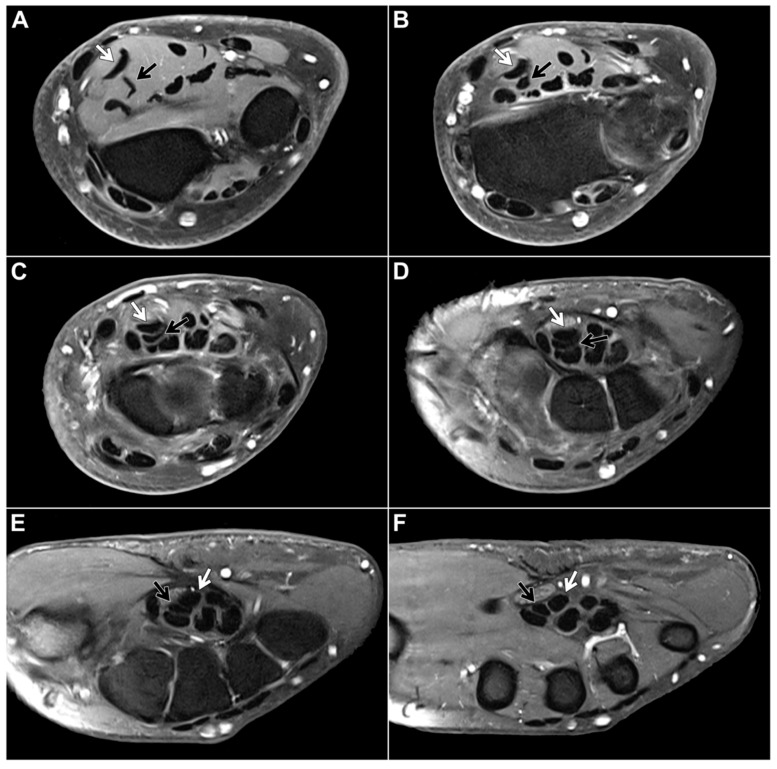
Fat-suppressed T1-weighted left forearm scan after intravenous administration of gadolinium-based contrast agent in a 48-year-old woman for presumed compression of the median nerve, which is located directly on the palmar side of the FDS tendons. Axial planes ((**A**,**B**): forearm level, (**C**–**E**): carpal tunnel level, (**F**): midcarpal level) depict the most common form of flexor digitorum superficialis anatomy: From the ulnar side of the forearm, the FDS/2 tendon (black arrow) crosses over the FDS/3 tendon (white arrow), thus forming the chiasma antebrachii.

**Figure 3:**
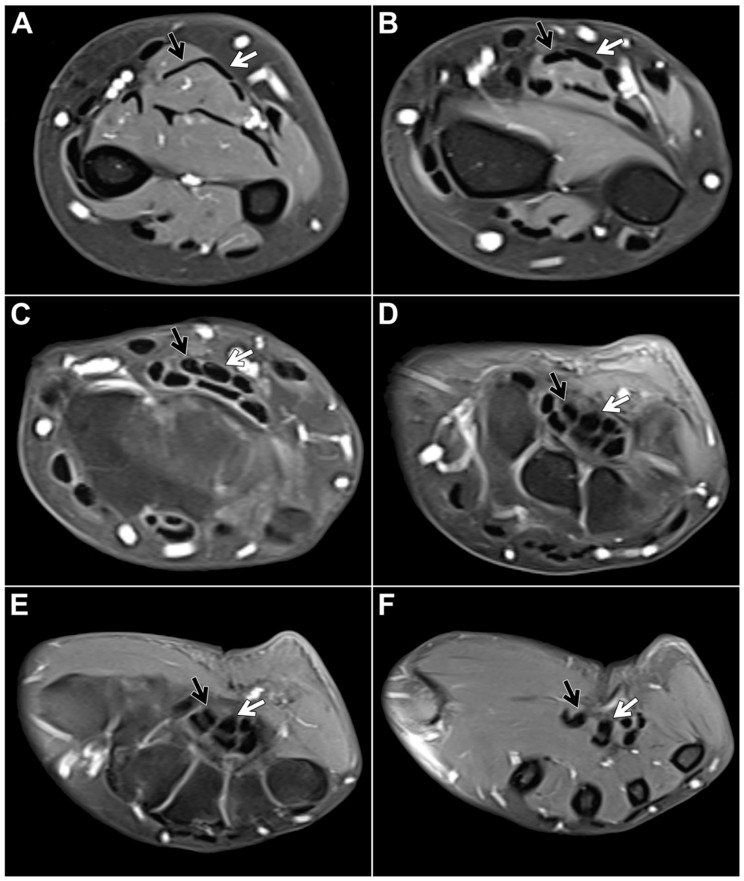
Contrast-enhanced, fat-suppressed T1-weighted turbo spin echo in a 44-year-old woman for presumed inflammatory disease of the right forearm depicts an anatomical variant of FDS anatomy without a typical (distal) chiasma antebrachii. Instead of an intersection, the distal FDS/2 (black arrow) and FDS/3 (white arrow) tendons run parallel to each other ((**A**,**B**): forearm level, (**C**–**E**): carpal tunnel level, (**F**): midcarpal level).

Within the study sample, the chiasma antebrachii with the above-described anatomical features was identified in 88 patients (98.9%). We defined the proximal margin of the chiasma as the first axial slice where the distal FDS/2 tendon was depicted inferiorly to the FDS/3 tendon and the distal margin as the first axial slice when the two tendons could be depicted horizontally adjacent to each other again (Figure 2). Measured according to these parameters, the chiasma antebrachii had a median length of 28 mm (IQR: 24 mm–35 mm). Next, we assessed the location of the chiasma in the forearm in relation to the distal radioulnar joint and the elbow joint. We measured the respective distance from the proximal margin and documented a median distance to the distal radioulnar joint of 16 mm (IQR: 8 mm–25 mm) and a median distance to the elbow joint of 215 mm (IQR: 187 mm–227 mm). Measurements were performed based on axial images but were correlated in at least one other standard plane. In only one patient, the intersection was located in the middle part of the forearm. In this particular case, the intermediate tendon of the FDS crossed under the FDS3 from the ulnar to the radial side. As a consequence, the distal FDS tendon segments for digits 2 and 3 could be depicted running parallel to each other in the distal third of the forearm up to their insertions at the mid phalanx of digits 2 and 3, with the FDS/2 located more radially (Figure 3).

Finally, the MRI sequence with the subjectively best depiction of the chiasma antebrachii was chosen by the observing radiologists. In 71 cases (79.8%), the observers deemed the T1-weighted post-contrast sequence superior, whereas a T2- or proton-density-weighted sequence was preferred in 18 examinations (20.2%).

### 3.3. Verification of Findings in Cadaveric Dissection Studies

**Figure 4:**
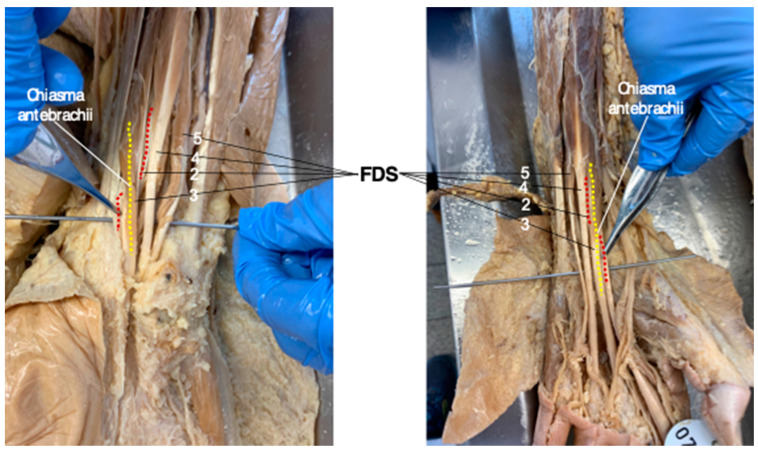
After careful preparation, the flexor digitorum superficialis (FDS) tendon anatomy is exposed in two body donates. In the dissected distal forearms of both cadaveric specimens (**left image**: right side; **right image**: left side), the chiasma antebrachii is marked by the FDS/2 tendon (highlighted in red) crossing under the FDS/3 tendon (highlighted in yellow). (2–5: FDS tendons for digit 2–5).

In all 11 human body donors assessed for this study, the chiasma antebrachii was present bilaterally in the distal portion of both forearms with the same anatomical configuration that was determined in the vast majority of MRI studies. Figure 4 shows the “standard” FDS tendon anatomy, including the chiasma antebrachii located in the distal portion of the forearm, in two cadaveric specimens (Figure 4).

## 4. Discussion

In this study, we provide a detailed MRI-based analysis of the recently introduced chiasma antebrachii, an anatomical feature defined by the distal FDS/2 tendon crossing under the FDS/3 from proximal-ulnar to distal-radial in the distal forearm. We were able to show that this intersection is typically localized in the distal portion of the forearm, where its presence could be determined in 98.9% of individuals. Only one intersection was found to be located more proximal in the middle part of the forearm. Based on our findings, we postulate that the chiasma antebrachii represents the standard anatomy of the flexor digitorum superficialis rather than an uncommon normal variant. In its standard configuration, it possesses a median length of 28 mm (IQR: 24 mm–35 mm).

The presented analysis of detailed MR imaging supports and confirms the anatomical description of the chiasma antebrachii as introduced by Ergün et al. based on anatomical dissection studies [16]. Our findings regarding the location of the chiasma concur with the observations in cadaveric specimens, as the distance of the tendon intersection to the elbow joint is reported at 220 ± 35 mm, while we measured a median distance of 215 mm. However, a distance to the wrist of 3–4 cm was described, as opposed to 16 mm, in this study. There are several possible explanations for this incongruity: First, we strictly defined the beginning of the chiasma antebrachii (and therefore our proximal measuring point) as the first axial plane where the FDS/2 tendon was depicted inferiorly to the FDS/3 tendon, whereas Ergün et al. measured the distance “from the tendon crossing”, rendering an exact comparison difficult. Second, we chose the distal radioulnar joint, a distinct landmark, as our distal measuring point to ensure reproducibility, while the transverse carpal ligament was used in the previous anatomical study [16]. As this ligament—with its osseous attachments to the scaphoid and trapezium on the radial side and the pisiform and hamate on the ulnar side—lies further distal of the distal radioulnar joint, the differing measurements seem plausible [20]. Lastly, measurements performed on 0.6–3 mm thick axial slices on a diagnostic monitor might be more accurate, especially in the millimeter range, compared to measuring by hand in dissection studies.

We deemed the best MRI sequence for the depiction of the chiasma antebrachii to be the T1-weighted post-contrast sequence, followed by a T2- or proton-density-weighted sequence. This result correlates with the latest protocol recommendations for the assessment of tendon-related pathologies of the distal forearm and hand in MRI [21,22]. To optimize image quality, it is also recommended that patients are placed prone with the arm extended overhead in the so-called “superman position”, which allows the placement of the wrist as close to the magnetic field’s isocenter as possible, therefore obtaining the highest signal-to-noise ratio and most homogeneous signal [22]. Adhering to these recommendations, the superman position constitutes the diagnostic standard in our department, with 82 (92.1%) of the scans analyzed in this study being performed this way. However, this position can be uncomfortable for some patients, rendering them unable to complete the examination. In these cases, other options may be considered, such as a supine position with the patient’s arm beside the body trunk or the “prayer position” with the patient flexing the elbow while lying on their side [22].

The exact understanding of the forearm muscle anatomy is important not only for teaching purposes in medical schools but also for clinical interventions such as operative tendon reconstruction, minimally invasive surgery, or target-specific application of therapeutics. For example, in cases of focal spasticity involving the upper limb, a precise injection of Botulinum neurotoxin into the affected muscles is considered the first-line treatment as this reduces spasticity while maintaining the motoric performance of the weakened spastic muscles [23,24]. Therefore, detailed knowledge of the functional and topographical anatomy of the FDS muscles, including awareness of the location and course of nearby structures, is needed to achieve a beneficial therapeutic outcome as well as to prevent accidental injections into unintended areas and reduce the risk of adverse events.

Muscle forces of the FDS (which is the strongest of the extrinsic hand muscles) are particularly strong during tip and lateral pinch motion, potentially inducing a higher degree of friction at the level of the chiasma. That being said, the chiasma antebrachii possesses similarities to the proximal (crossing of the abductor pollicis longus and extensor pollicis brevis tendons over the extensor carpi radialis longus and brevis tendons) and distal extensor tendon intersections (crossing of the extensor pollicis longus tendon over the extensor carpi radialis longus and brevis tendons) on the dorsal side of the wrist [21,25]. The two listed tendon intersections are of great clinical relevance: repetitive extension–flexion movements at these junctions commonly seen in sporting activities can result in a localized friction injury, generating tenosynovitis and characteristically leading to pain, swelling, and functional limitations—a clinical condition termed “intersection syndrome” [26,27]. Considering the anatomic similarities, the occurrence of a clinically relevant flexor-sided intersection syndrome in patients with intensive use of the index and middle fingers is conceivable. While future studies should investigate whether the FDS tendons in symptomatic patients display signs of friction-induced tenosynovitis at the level of the chiasma, our work suggests that MRI may be a suitable technique for assessing suchlike patients in clinical routine.

Some limitations have to be acknowledged with regard to this study. The retrospective design of the study resulted in a heterogeneous patient population in terms of sociodemographic data and clinical history. Factors such as tumor presence in the forearm, history of trauma, and soft tissue inflammation introduced variations among the participants. However, these pathologies did not have a significant impact on the specific questions addressed in this study. This is primarily because the respective conditions did not exhibit a direct topographical relationship with the FDS tendons under examination. Therefore, despite the heterogeneity within the patient population, the findings of the study remain valid and relevant to the research questions posed. Additionally, the inclusion of patients with various sociodemographic backgrounds and clinical histories contributes to the generalizability of the study findings to a broader population. As the scans were performed for different clinical indications, the scan protocols varied depending on the initial clinical question. Scans with a slice thickness of 0.6 mm might allow for a more precise evaluation and measurement than protocols with a greater slice thickness.

With this study, we provide new insights into the complex anatomy of the flexor digitorum superficialis with a specific focus on the chiasma antebrachii. Our results not only support the initial description of the chiasma by Ergün et al. but also emphasize its significance as a key anatomic feature that can be observed in nearly all individuals. Furthermore, this research might serve as a foundation for future studies exploring the clinical implications of the chiasma antebrachii in conditions such as intersection syndromes.

## Data Availability

The datasets generated and/or analyzed during this study are not publicly available, as MRI data and DICOM headers contain patient information. Data can be obtained on reasonable request from the corresponding author.
